# Membranous Nephropathy Target Antigens Display Podocyte-Specific and Non-Specific Expression in Healthy Kidneys

**DOI:** 10.3390/genes16030241

**Published:** 2025-02-20

**Authors:** Ying Dong, Hui Xu, Damu Tang

**Affiliations:** 1 Department of Surgery, McMaster University, Hamilton, ON L8S 1C7, Canada; dongy87@mcmaster.ca; 2 Urological Cancer Center for Research and Innovation (UCCRI), St Joseph’s Hospital, Hamilton, ON L8N 4A6, Canada; 3 The Research Institute of St Joe’s Hamilton, St Joseph’s Hospital, Hamilton, ON L8N 4A6, Canada; 4 The Division of Nephrology, Xiangya Hospital of the Central South University, Changsha 410008, China; xuhuiye@csu.edu.cn

**Keywords:** membranous nephropathy, MN target antigens, podocyte, single-cell RNA sequencing

## Abstract

Background/Objectives: Autoimmunity towards podocyte antigens causes membranous nephropathy (MN). Numerous MN target antigens (MNTAgs) have been reported, including PLA2R1, THSD7A, NTNG1, TGFBR3, HTRA1, NDNF, SEMA3B, FAT1, EXT1, CNTN1, NELL1, PCDH7, EXT2, PCSK6, and NCAM1, but their podocyte expression has not been thoroughly studied. Methods: We screened CZ CELLxGene single-cell RNA (scRNA) sequence datasets for those of adult, fetal, and mouse kidneys and analyzed the above MNTAgs’ expression. Results: In adult kidneys, most MNTAgs are present in podocytes, except PCSK6 and NCAM1. PLA2R1 is expressed significantly more than other MNTAgs in podocytes and is a major podocyte marker, consistent with PLA2R1 as the dominant MNTAg. Additionally, PLA2R1 is a top-upregulated gene in the podocytes of chronic kidney disease, acute kidney injury, and diabetic nephropathy, indicating its general role in causing podocyte injury. PLA2R1, NTNG1, HTRA1, and NDNF display podocyte-enriched expression along with elevated chromatin accessibility in podocytes, suggesting transcription initiation contributing to their preference expression in podocytes. In the fetal kidney, most MNTAgs are expressed in podocytes. While PLA2R1 is weakly present in podocytes, SEMA3B is abundantly expressed in immature and mature podocytes, supporting SEMA3B as a childhood MNTAg. In mouse kidneys, Thsd7a is the only MNTAg with a prominent level and podocyte-specific expression. **Conclusions**: Most MNTAgs are present in podocytes in adults and during renal development. In adults, PLA2R1 expression is highly enriched in podocytes and significantly upregulated in multiple kidney diseases accompanied by proteinuria. In mouse kidneys, Thsd7a is specifically expressed in podocytes at an elevated level.

## 1. Introduction

Membranous nephropathy (MN) is a major cause of adult nephrotic syndrome [1,2] and can progress to end-stage kidney disease (ESKD) in one-third of patients without therapeutic interventions [3,4,5]. MN is an autoimmune disease of the kidney with hallmark deposition of IgG and complements within the glomerular subepithelial space [3]; the immune complex is largely formed by circulating autoantibodies reacting to specific antigens expressed in podocytes. This notion was validated owing to the establishment of Heymann nephritis rats in the 1950s [6] and the subsequent identification of megalin or GP330 as the target antigen in 1982 [7], as well as the discovery of PLA2R1 as the major target antigen in MN patients in 2009 [8]. GP330 is exclusively expressed in rat podocytes [9]; a clear expression of PLA2R1 in human podocytes was also demonstrated [8]. In both Heymann nephritis rats and patients with PLA2R1-related MN, autoantibodies recognizing the target antigens were present in circulation [6,7,8]. Recently, numerous MN target antigens (MNTAgs) were identified, including THSD7A [10], NTNG1 [11], TGFBR3 [12], HTRA1 [13], NDNF [14], SEMA3B [15], FAT1 [16], EXT1/2 [17], CNTN1 [18], NELL1 [19], PCDH7 [20], PCSK6 [21], and NCAM1 [22]. While these MNTAgs can be detected in primary MN (PMN) cases lacking other diseases [23,24,25], they are more often associated with secondary MN (SMN) with underlying diseases [23,24]. In comparison, PLA2R1 is mainly associated with PMN, which accounts for approximately 80% of all MN [3,26]. Circulating autoanti-PLA2R1 antibodies are detected in 80% of PMN cases [3,8,27]. PLA2R1 is thus the major MNTAg and the rest of the MNTAgs represent small proportions of MN cases [23,24]. Nonetheless, PLA2R1 and most other MNTAgs share similarities with respect to their expression in podocytes and the presence of circulating antibodies, revealing the pathological importance of podocyte injury in MN occurrence. However, their podocyte expression, including PLA2R1, in the context of the kidney, has not been systemically examined, let alone their expressions in podocytes at the single-cell level. In view of the direct and dominant role of podocyte injury in MN pathogenesis, the detailed knowledge of MNTAg expression in the kidney and podocyte is important not only in advancing our understanding of MN but also in further investigating the mechanisms underlying MN pathogenesis.

We report a thorough analysis of PLA2R1, THSD7A, NTNG1, TGFBR3, HTRA1, NDNF, SEMA3B, FAT1, EXT1, CNTN1, NELL1, PCDH7, EXT2, PCSK6, and NCAM1 expressions in podocytes in the context of the kidney using the available n = 10 single-cell RNA sequencing (scRNAseq) datasets, including eight adult, one fetal, and one mouse kidney datasets. We observed PLA2R1 as a novel podocyte-specific marker. Additionally, NTNG1, HTRA1, and NDNF show a clear preference for podocyte expression. The rest of the MN antigens do not show podocyte preference expression. In human podocytes, PLA2R1 displays a dominant expression among all 15 MNTAgs. In comparison, Thsd7a is the only MNTAg that is abundantly and specifically expressed in mouse podocytes, consistent with Thsd7a being commonly used in mouse models for MN. Based on the expression index in human podocytes, the 15 MNTAgs can be separated into high, medium, low, and non (+/−)-expression groups. The knowledge obtained in this study will facilitate further MN research.

## 2. Materials and Methods

### 2.1. Data Source

The single-cell RNA sequencing (scRNAseq) data platform CZ CELLxGENE Discover (https://cellxgene.cziscience.com/, accessed on 25 December 2024) [28] was used. Under the “Collections” function (https://cellxgene.cziscience.com/collections, accessed on 25 December 2024), we screened scRNA datasets using the criteria of “Normal, Homo sapiens, renal system, kidney, and cortex of kidney” (27 December 2024); a total of 9 studies/collections were selected, including Laket et al. (2023) [29], “HCA kidney seed network: University of Michigan” (https://cellxgene.cziscience.com/collections/a98b828a-622a-483a-80e0-15703678befd, accessed on 27 December 2024), Xu et al. (2023) [30], McEvoy et al. (2022) [31], Wilson et al. (2022) [32], Marshall et al. (2022) [33], Muto et al. (2021) [34], Stewart et al. (2019) [35], and Young et al. (2018) [36]. The McEvoy et al. dataset contained only n = 16 podocytes and was thus excluded from further analysis. We also analyzed the only mouse kidney scRNA dataset, Novella-Rausell et al. (2023) [37], which was available in the CZ CELLxGENE Discover platform.

### 2.2. Quantification of MN Target Antigen Expression in Podocytes

We utilized the “Gene Expression” function within the CZ CELLxGENE Discover platform to analyze the podocyte expression of the MNTAgs PLA2R1, THSD7A, NTNG1, TGFBR3, HTRA1, NDNF, SEMA3B, FAT1, EXT1, CNTN1, NELL1, PCDH7, EXT2, PCSK6, and NCAM1 in human kidney. These gene expressions in human kidneys were scaled. The gene expression index for individual genes was calculated by the scaled gene expression × % of gene expression in podocyte × 100.

### 2.3. Differential Gene Expressions in Human Podocytes

Using the “Differential Expression” function within the CZ CELLxGENE Discover platform, we determined podocyte gene expression between normal and chronic kidney disease (CKD), acute kidney injury, or diabetic nephropathy from type 2 diabetes mellitus.

## 3. Results

### 3.1. Podocyte-Specific Expression of PLA2R1

**Figure 1:**
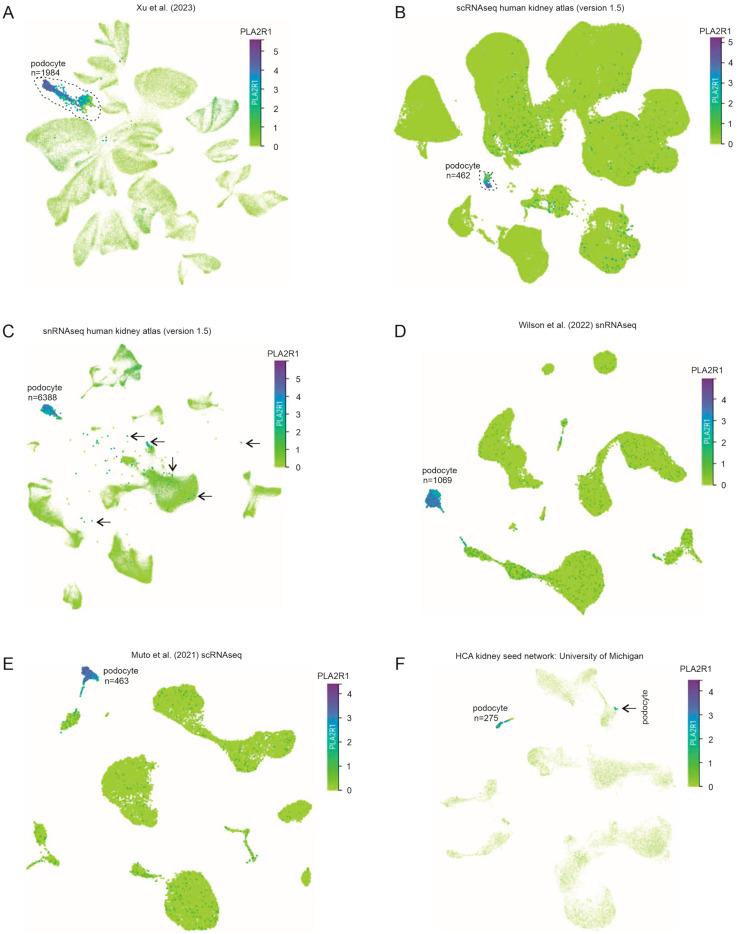
Dominant PLA2R1 expression in podocytes. PLA2R1 mRNA expression in single-cell populations of human kidneys within the following datasets: (**A**) Xu et al. (2023) [30], (**B**) scRNAseq human kidney atlas (version 1.5), (**C**) snRNAseq human kidney atlas (version 1.5), (**D**) Wilson et al. (2022) snRNA [32], (**E**) Muto et al. (2021) scRNAseq [34], and (**F**) HCA kidney seed network: University of Michigan. The podocyte clusters are indicated. For the identities of other kidney cell clusters, please see Supplementary Figures S1–S4. (**A**,**C**,**F**) Podocytes are gated to show PLA2R1 expression. Arrows indicate podocytes (**C**).

Immunofluorescence staining detected abundant PLA2R1 protein expression in human podocytes [8]; Northern Blot analysis revealed the kidney as the major tissue expressing PLA2R1 mRNA [38]. However, as the major MN target antigen in adult patients [8], PLA2R1 expression in the kidney has not been systematically studied. Given the critical role of podocyte injury in MN pathogenesis, we thought to, in detail, analyze PLA2R1 expression in the kidney at the single-cell level. CZ CELLxGENE Discover has a comprehensive collection of scRNA data in multiple species and across both normal and disease conditions [28]. We selected all scRNA datasets (n = 9) relevant to “Normal”-“Homo sapiens”-“renal system”-“kidney”-“cortex of kidney” within CZ CELLxGENE Discover and excluded the McEvoy et al. dataset as it contained only 16 podocytes. The single cell map from five human kidneys within the Xu et al. (2023) dataset [30] was downloaded and re-labeled (Supplementary Figure S1A). The podocyte cluster was confirmed by showing the dominant expression of the well-established podocyte markers nephrin (NPHS1) (Supplementary Figure S1C), WT-1 (Wilms’ tumor 1), and podocin (NPHS2). PLA2R1 displays a similar expression pattern to NPHS1 (Supplementary Figure S1B), with a broader weak expression in other kidney cells (comparing Supplementary Figure S1B to Supplementary Figure S1C). Nonetheless, PLA2R1 is expressed at a slightly higher level than NPHS1 in podocytes, based on the scaled bar and intensity of both gene expressions (comparing Supplementary Figure S1B to Supplementary Figure S1C), which might contribute to a wider distribution of PLA2R1 weak expression in other kidney cells compared to NPHS1. By gated podocytes, the high level of PLA2R1 expression in these cells in the Xu et al. (2023) dataset is apparent (Figure 1A). By using the scRNAseq and single nuclear RNAseq (snRNAseq) human kidney atlas (version 1.5) datasets, both of which were derived from the Laket et al. (2023) study [29], dominant podocyte expression of PLA2R1 was demonstrated (Figure 1B,C; Supplementary Figure S2). We noticed that cells with high PLA2R1 expression outside of the podocyte cluster (Supplementary Figure S2D) were podocytes, evidenced by their positivity when kidney cells were gated on podocytes (Figure 1C). The dominant podocyte expression of PLA2R1 was also revealed in the Wilson et al. scRNAseq, Muto et al. scRNAseq, and HCA kidney seed network: University of Michigan datasets (Figure 1D–F, Supplementary Figures S3 and S4). In comparison to NPHS1, PLA2R1 was also mainly expressed in podocytes in both the Young et al. (2018) [36] and Stewart et al. (2019) [35] datasets (Supplementary Table S1).

**Figure 2:**
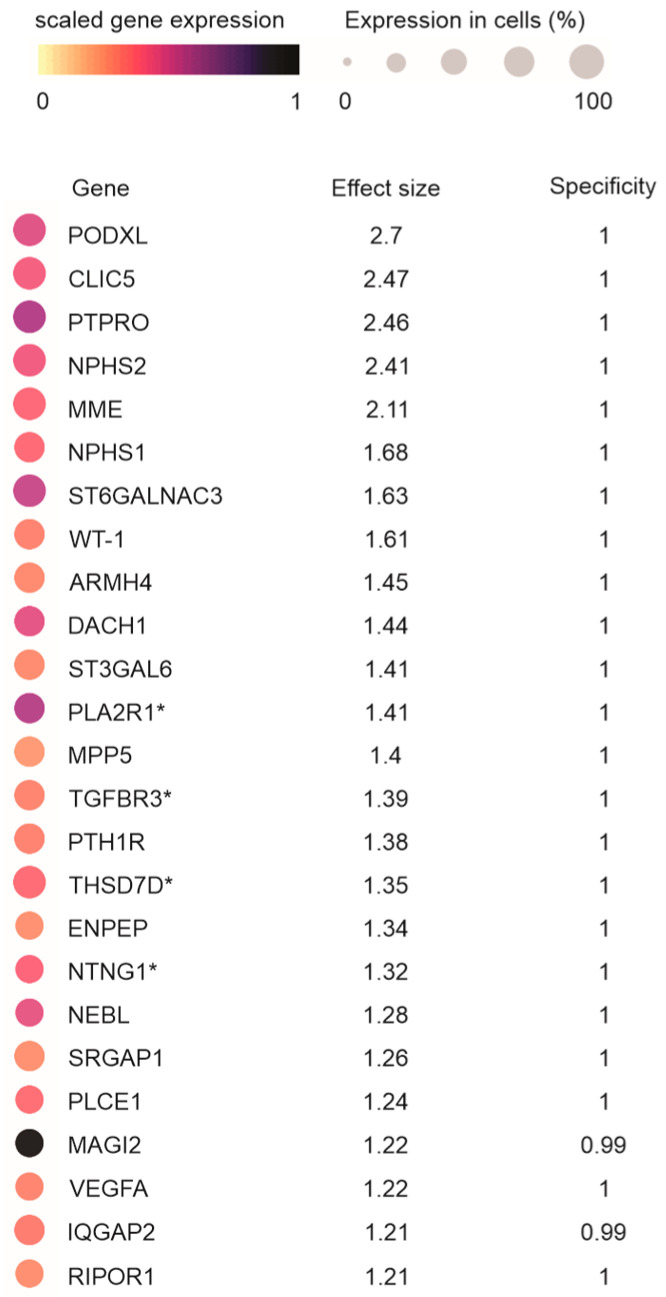
PLA2R1 as a podocyte marker. Podocyte marker genes in the human kidney were analyzed. The top 25 podocyte marker gene expressions were determined at the single-cell level using 1.1 million kidney cells and 13,100 podocytes from 20 datasets within CZ CELLxGENE Discover. The effect size and specificity associated with their podocyte expressions are included. *: MN target antigens.

To further analyze the specificity of PLA2R1 expression in podocytes, we downloaded podocyte marker genes from CZ CELLxGENE Discover (https://cellxgene.cziscience.com/gene-expression, accessed on 27 December 2024), which was determined from 20 single-cell datasets containing 1.1 million human kidney cells and 13,100 podocytes. Ranked by the effect size, PLA2R1 is the top twelfth podocyte marker gene with an effect size of 1.41 and specificity of 1 (Figure 2). Among the top 25 podocyte marker genes are well-established podocyte markers, including podocin (NPHS2), nephrin (NPHS1), and WT-1 (Figure 2). Both podocin and nephrin are podocyte slit diaphragm proteins; mutations in either protein cause podocyte injury, leading to early-onset proteinuria [39,40]. WT-1 is a master transcription factor of podocytes [41]. Interestingly, the TGFBR3, THSD7A, and NTNG1 MNTAgs also exhibit podocyte preference expression (Figure 2), supporting the important pathological contributions of podocyte injury to the kidney-limited autoimmune disease of MN.

### 3.2. Podocyte Expression of MN Target Antigens

**Figure 3:**
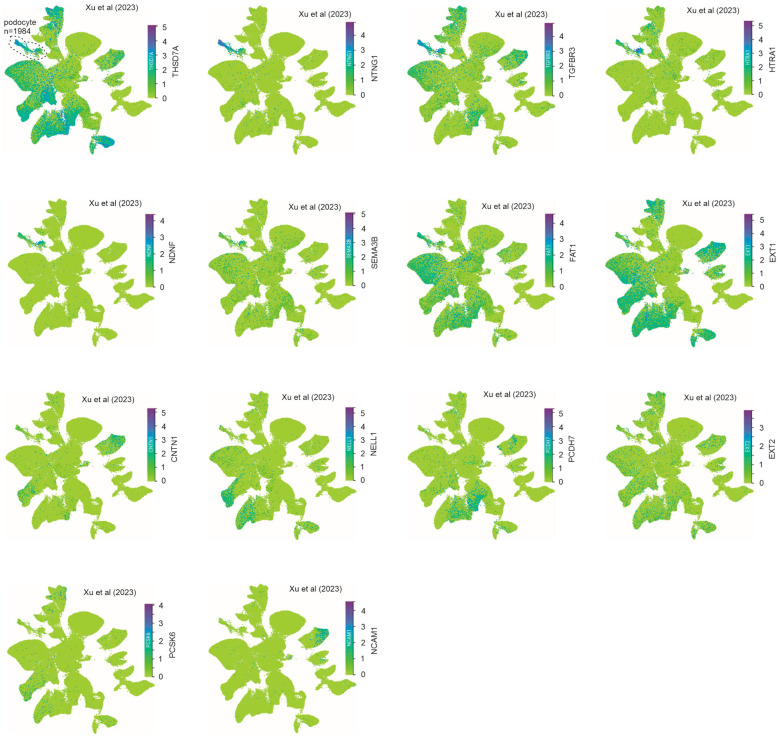
Expression of MNTAgs in podocytes and other kidney cells. The kidney expression of the indicated MN target antigens was determined in the Xu et al. (2023) [30] dataset within the CZ CELLxGENE Discover platform. The podocyte cluster is indicated. For the identities of other kidney cell clusters, please see Supplementary Figure S1A.

We then examined the expressions of other MNTAgs, THSD7A, NTNG1, TGFBR3, HTRA1, NDNF, SEMA3B, FAT1, EXT1/2, CNTN1, NELL1, PCDH7, PCSK6, and NCAM1, in podocytes. In the Xu et al. (2023) dataset, we observed high-level expressions of THSD7A, NTNG1, and TGFBR3 in podocytes (Figure 3), supporting their preference expression in podocytes (Figure 2); medium-level expression of HTRA1, NDNF, SEMA3B, FAT1, and EXT1 in podocytes (Figure 3); low podocyte expression of CNTN1, NELL1, PCDH7, and EXT2 (Figure 3); and a substantially low-level expression of PCSK6 and NCAM1 in podocytes (Figure 3). Similar observations were also obtained from the Wilson et al. snRNAseq dataset (Supplementary Figure S5), as well as the Youg et al. (2018), Stewart et al. (2019) (Supplementary Table S1), and other datasets. Among these MNTAgs, NTNG1, HTRA1, and NDNF exhibit podocyte preference in both the Xu et al. (2023) and Wilson snRNA datasets (Figure 3, Supplementary Figure S5).

**Figure 4:**
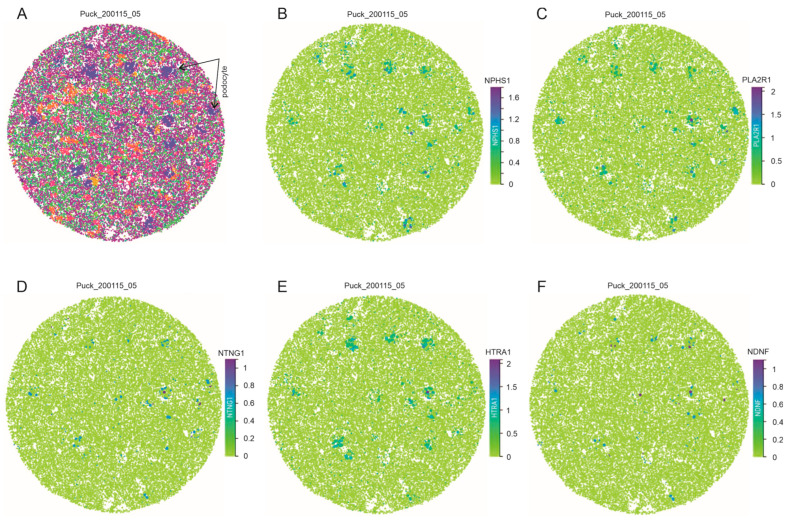
Expression of PLA2R1, NTNG1, HTRA1, and NDNF in situ within the podocyte clusters. The snRNAseq dataset derived from human kidney slides by Marshall et al. (2022) [33] was used here. Podocytes are highlighted within the kidney cell map (**A**); the expression of the indicated genes in the kidney tissues is shown (**B**–**F**). NPHS1 is used as a control for podocytes.

We subsequently examined podocyte expression of PL2AR1, NTNG1, HTRA1, and NDNF at a single-cell level using human kidney slides from the Marshall et al. (2022) dataset [33] by taking advantage of the preservation of the location information of gene expression. In the slide Puck_200115_05, we downloaded and reproduced the cell map image with podocyte highlighted (Figure 4A); the podocyte clusters indicate the presence of glomeruli (Figure 4A). As a podocyte marker, NPHS1 expression is within the podocyte clusters, confirming that the mapped cells are podocytes (Figure 4B). PLA2R1, NTNG1, HTRA1, and NDNF are all expressed within individual podocyte clusters (Figure 4C–F). Similar observations were also obtained using a kidney slide from another human kidney, Puck_200104_20 (Supplementary Figure S6), and three other human kidney slides, Puck_200113, Puck_200131, and Puck_200903. Collectively, we provide comprehensive analyses for the podocyte expression of most reported MNTAgs. The podocyte-focused expression of PLA2R1, NTNG1, HTRA1, and NDNF is intriguing, highlighting antigen-initiated autoimmunity as causing podocyte injury and the subsequent MN pathogenesis.

### 3.3. Variation of MN Target Antigens’ Expression in Podocytes

**Figure 5:**
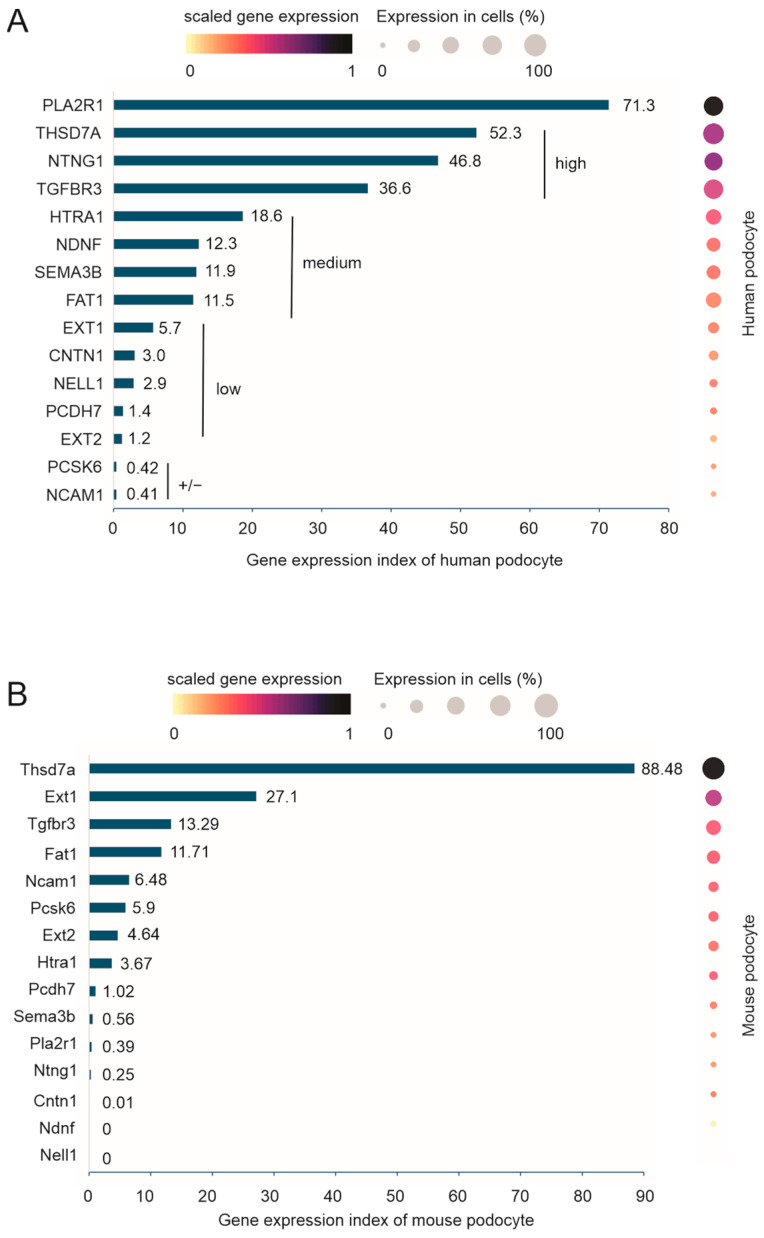
Quantification of MNTAgs’ expression in human and mouse podocytes. (**A**) Quantification of the indicated MN target antigen expression using 13,074 podocytes in 20 datasets within the CZ CELLxGENE Discover platform. Gene expression index was determined using gene expression, scaled according to the expressions of the indicated MNTAgs as a group, and the percentage of podocytes expressing individual MNTAgs following the formula: scaled gene expression × % of podocytes expressing the relevant gene × 100. Genes are categorized into high-, medium-, low- or possibly non (+/−)-expressing groups. (**B**) Quantification of the murine counterparts of human MNTAgs’ expression in mouse podocytes using one dataset containing 469 podocytes.

With the demonstration of the podocyte expression of at least 15 MNTAgs (PLA2R1, THSD7A, NTNG1, TGFBR3, HTRA1, NDNF, SEMA3B, FAT1, EXT1, EXT2, CNTN1, NELL1, PCDH7, PCSK6, and NCAM1), it will be interesting to know their level of expression in podocytes, which might be relevant to their ability to cause MN. To address this question, we quantified their expression in podocytes by inputting these antigens as a group into the “Gene Expression” function within CZ CELLxGENE Discover and then retrieved scaled gene expression, which was produced based on the expression of these antigens, and the percentage of podocytes in which individual antigens are expressed. The scaled gene expression allows for a comparison of gene expressions among these antigens. We then calculated the expression indices for individual genes using the formula: scaled gene expression × % of podocytes expressing the target gene × 100. PLA2R1 displays a dominantly high-level expression in podocytes (Figure 5A), which might be an underlying mechanism for PLA2R1 as the dominant MNTAgs [8,24,42]. Other than PLA2R1, THSD7A, NTNG1, and TGFBR3 are expressed at high levels in podocytes (Figure 5A); HTRA1, NDNF, SEMA3B, and FAT1 can be grouped in MNTAgs with medium expression in podocytes (Figure 5A); EXT1, CNTN1, NELL1, PCDH7, and EXT2 are separated into the group of MNTAgs with low levels of podocyte expression (Figure 5A); and PCSK6 and NCAM1 may not be expressed in podocytes (Figure 5A), consistent with reports of both being not expressed in podocytes [43].

We also analyzed the mouse counterparts of the above MNTAgs’ expression in mouse podocytes. In the only mouse scRNAseq dataset containing 469 podocytes, Novella-Rausell et al. (2023) [37], Thsd7a is the only gene with a high level of podocyte-specific expression (Supplementary Figure S7) and exhibits a dominantly high-level expression in mouse podocytes (Figure 5B), supporting that mice are an effective passive MN model for THSD7A-caused MN [44]. Based on the level of MNTAg expression in podocytes, Ext1, Tgfbr3, Fat1, possibly Ncam1, Pcsk6, Ext2, and Htra1 (Figure 5B) might be useful target antigens to be explored for MN pathogenesis in mice via either passive antibody transfer or vaccination. However, both mouse MN models are likely not feasible for Pcdh7, Sema3b, Pla2r1 (as demonstrated), Ntng1, Cntn1, Ndhf, and Nell1 (Figure 5B).

### 3.4. Association of Chromatin Accessibility with Podocyte Preference Expression of MNTAgs

**Figure 6:**
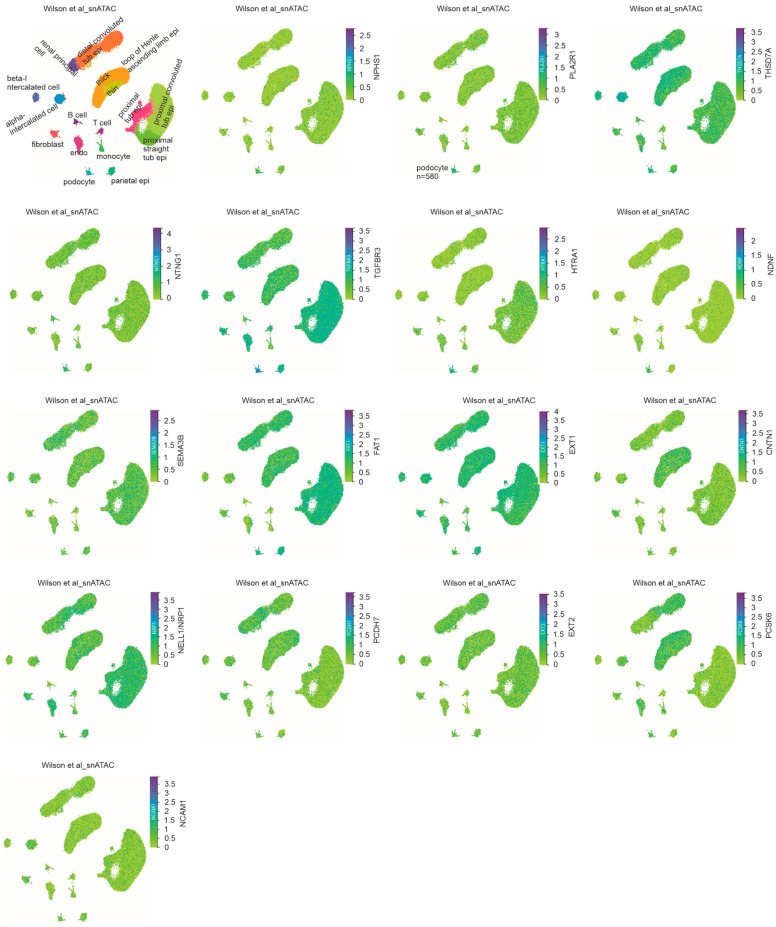
Chromatin accessibility of genes encoding for MNTAgs in podocytes and other kidney cells. The snATAC dataset from the Wilson et al. (2022) dataset [32] was used here. The cell map was reproduced (top left panel). Chromatin accessibility for the indicated genes in podocytes and other kidney cell populations is shown.

We studied the regulations contributing to MNTAg expression in podocytes. By taking advantage of the available snATAC (assay for transposase-accessible chromatin) dataset, Wilson et al. snATAC [32] containing 580 podocytes (Figure 6, cell map), we detected a slightly higher level of accessible chromatin for the *NPHS1* gene in podocytes compared to other kidney cell populations (Figure 6), validating the utility of the dataset in analyzing the level of transcription initiation, which is associated with chromatin accessibility. All genes encoding the MNTAgs examined here are associated with open chromatin in podocytes (Figure 6); the chromatin of the *PCDH7*, *EXT2*, *PCSK6*, and *NCAM1* genes is opened to a lower level (Figure 6), consistent with their low mRNA abundance in podocytes (Figure 5A). The *PLA2R1*, *NTNG1*, *HTRA1*, and *NDNF* genes have higher levels of chromatin opening in podocytes compared to other kidney cells (Figure 6), suggesting the enhanced initiation of transcription contributing to their preference expression in podocytes.

### 3.5. Developmental Expression of MN Target Antigens

MNTAgs are associated with MN occurrence in different age groups. While PLA2R1 is the dominant MN target antigen in adult patients with a mean age of 58 years [8], SEMA3B mainly affects pediatric MN patients, with disease onset starting at or below 2 years old in most patients [15]. We thus analyzed MNTAg expression at the developmental stage from >56 days to birth using the Steward et al. (2019) dataset [35]. During kidney development, some epithelial precursor cells in the proximal segment of the S-shaped body differentiate into podocytes [45]; these podocyte precursor cells or immature podocytes express podocalyxin (encoded by PODXL) [46], the tight junction protein ZO-1 (encoded by TJP1) [47], and the master podocyte transcription factor WT-1 [48,49]. WT-1 is one of the earliest podocyte markers expressed during renal development [50]. In line with this knowledge, PODXL, ZO-1, and WT-1 are mainly expressed in proximal S-shaped bodies and podocytes in fetal kidneys (Supplementary Figure S8), providing a validation of the fetal kidney scRNA dataset [35].

**Figure 7:**
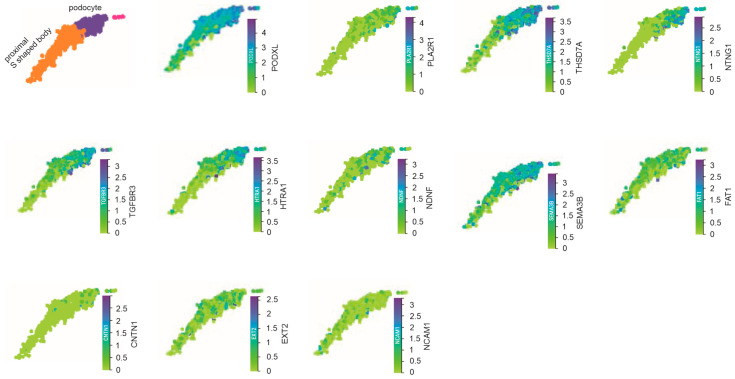
Expression of MNTAgs in podocytes during renal development. Analysis was performed using the scRNA dataset from a fetal kidney at the developmental stages from day 56 onward to birth (CELLxGENE) [35]. The proximal S-shaped body and podocyte clusters are indicated. The expressions of PODXL as a positive control for immature and mature podocytes and the indicated MNTAgs are shown.

While the MNTAgs EXT1, NELL1, PCDH7, and PCSK6 exhibit no detectable expression in the proximal S-shaped body and podocytes, PLA2R1, THSD7A, NTNG1, TGFBR3, HTRA1, NDNF, SEMA3B, and FAT1 are expressed in these cell clusters in the fetal kidney (Figure 7, Supplementary Figures S8 and S9). In contrast with FAT1, CNTN1, EXT2, and NCAM1, which are present in substantially low levels in fetal podocytes, PLA2R1, THSD7A, NTNG1, TGFBR3, and HTRA1 display a preference for podocyte expression in fetal podocytes (Supplementary Figures S8 and S9). For those MNTAgs expressed in fetal podocytes, PLA2R1, THSD7A, NTNG1, TGFBR3, HTRA1, NDNF, and CNTN1 are mainly expressed in mature podocytes compared to immature podocytes within the proximal S-shaped body (Figure 7). Different from its abundant expression in adult podocytes (Figure 5A), PL2AR1 is expressed at a low level in fetal kidneys (Figure 7), consistent with PL2AR1 as the major MN antigen in adult MN patients [8]. SEMA3B is expressed at a high level in immature (proximal S-shaped body) and mature podocytes (Figure 7), supporting it as a pediatric MN antigen [15]. As one of the two most-studied MNTAgs, THSD7A is expressed abundantly in both adult and fetal podocytes (Figure 5A, Figure 7) with podocyte-specific expression in the fetal (Supplementary Figure S8) but not the adult kidney (Figure 3). Taken together, we provide an initial analysis of the developmental expression of MNTAgs.

### 3.6. PLA2R1 as a Top-Upregulated Gene in Chronic Kidney Disease (CKD), Acute Kidney Injury (AKI), and Diabetic Nephropathy (DN) from Type 2 Diabetes Mellitus

Podocyte injury is the key pathological factor leading to proteinuria, including diabetic nephropathy (DN) and other proteinuric glomerular diseases [49,51,52]. Proteinuric glomerular diseases contribute to 80% of cases progressing to end-stage kidney disease (ESKD) [53]. The loss of podocytes is a hallmark of developing kidney failure [49,54]. Given that most MNTAgs are expressed in podocytes and directly contribute to podocyte injury in the respective MN diseases, we speculated their involvement in other kidney diseases associated with proteinuria, including CKD and DN. Evidence from animal studies [55] and comprehensive clinical research [56] revealed that AKI contributes to the development and worsening of proteinuria, suggesting a possible involvement of MNTAgs in AKI-associated proteinuria. We comprehensively analyzed eleven scRNA datasets with 8257 normal podocytes, three scRNA CKD datasets with 2946 podocytes, three AKI datasets with 1471 podocytes, and one type 2 diabetes mellitus dataset with 390 podocytes and detected PLA2R1 as being in the top two or three upregulated genes in the comparison settings of CKD to normal, AKI to normal, and DN to normal (Table 1).

Among the upregulated genes, MAGI2 (membrane-associated guanylate kinase, WW, and PDZ domain containing 2) mutation is a cause of the nephrotic syndrome [57]. The long non-coding RNA (lncRNA) FTX displays activities in promoting renal cell carcinoma proliferation [58]; its upregulation may re-initiate podocyte proliferation, which will damage podocytes. PARD3B (Par-3 family cell polarity regulator β) is expressed in the slit diaphragm and was suggested to play a role in the filtration function of the glomerular filtration barrier [59]. Alterations in other genes in this gene list in kidney disease are unclear. Nonetheless, the identification of PLA2R1 as a top-upregulated gene in CKD, AKI, and DN suggests that PLA2R1 may play a general role in causing podocyte injury, which contributes to proteinuria in these kidney diseases. This concept is intriguing, considering the unknown mechanisms leading to the subsequent occurrence and worsening proteinuria following AKI [56].

## 4. Discussion

Since the identification of PLA2R1 as the major MNTAg in 2009 [8], at least 14 other MNTAgs were lately reported, including THSD7A, NTNG1, TGFBR3, HTRA1, NDNF, SEMA3B, FAT1, EXT1, CNTN1, NELL1, PCDH7, EXT2, PCSK6, and NCAM1 [24]. Cumulative data reveals a significant role of MNTAgs in directly causing podocyte injury. The relevance of MNTAgs in MN pathogenesis is reflected by the recent call for a two-step MN clarification by the Mayo Clinic consensus group [60], which consists of the first-step clarification according to MNTAgs and the second-step determination of the underlying conditions. As knowledge of MNTAgs accumulates, it is crucial to deepen our understanding of their expressions in podocytes in the context of the kidney. This research contributes to this aim by providing a first and comprehensive analysis of MNTAg expression at the single-cell level. Our systemic study of MNTAgs’ expression would enhance antigen-based MN clarification.

We demonstrated that most of the 15 MNTAgs (PLA2R1 plus the other 14 MNTAgs above) are present in podocytes in healthy human kidneys (Figure 5A). PCSK6 and NCAM1 may not be expressed in podocytes, which is consistent with the existing knowledge [43]. MNTAg genes are expressed at various levels and can be divided into individual groups according to their levels of expression in podocytes (Figure 5A). Their expression level might be relevant to their ability to cause MN autoimmunity towards podocytes. We report that PLA2R1 is expressed at a higher level in podocytes than other MNTAgs (Figure 5A), which might be partly attributable to PLA2R1 as the major MN target antigen [8]. Nonetheless, other mechanisms are clearly in place and affect MN pathogenesis. For instance, genome-wide association studies (GWASs) revealed multiple single-nucleotide polymorphisms (SNPs) in the *PLA2R1* gene, with SNP rs4664308 displaying the most significant association (*p* = 8.6 × 10^−29^) [61]. Given that the complex mechanisms are involved in MN, the knowledge of MNTAgs’ expressions, obtained in this investigation, might be utilized to decipher these pathogenic mechanisms and the disease onset in the future.

Despite the major pathological involvement of PLA2R1 in MN, the underlying mechanisms remain largely unknown, which is partially attributable to the absence of the physiological roles of PLA2R1. Mice deficient in *Pla2r1* are healthy [62] and mouse podocytes do not express Pla2r1 (Figure 5B). The potentially non-essential role of PLA2R1 in overall human health might explain why this gene is specifically associated with membranous nephropathy (PLA2R1-MN) but not linked to a broader range of diseases. In this context, the silencing of PLA2R1 in podocytes might be a therapeutic avenue to be explored in the future in managing patients with PLA2R1-positive MN who are at risk of ESKD progression.

We provide the first evidence for low PLA2R1 expression in podocytes during renal development (Figure 7; Supplementary Figure S8), supporting its role as a target antigen in adult MN cases. In contrast, SEMA3B is abundantly and specifically expressed in developing podocytes (Figure 7; Supplementary Figure S9), which may partially explain its candidacy as an MN target antigen in children.

In addition to PLA2R1, we detected significant enrichment in the expression of NTNG1, HTRA1, and NDNF in podocytes. This enrichment is associated with a higher level of chromatin accessibility in podocytes (Figure 6), suggesting that transcription initiation is a factor for their preference expression in podocytes. This knowledge may be relevant in developing therapies targeting the mechanisms regulating the chromatin opening for these genes in managing the respective MN patients.

This research provides the first detailed insights into MNTAg expression in mouse podocytes. Interestingly, Thsd7a is likely expressed in mouse podocytes specifically at a prominent level (Figure 5B, Supplementary Figure S7), providing an explanation as to why the Thsd7a-based mouse MN model displays a rapid onset of MN and has been widely used in MN research [44]. Ext1, Tgfbr3, and Fat1 might be useful in mouse models for MN; however, the disease onset might be slow owing to their low-level expression in mouse podocytes (Figure 5B). Additionally, Pla2r1, Pcdh7, Sema3b, Ntng1, Cntn1, Ndnf, Nell1, Pcsk6, Ext2, and Htra1 may not be feasible to be used in mice for either passive antibody transfer or vaccination-based MN investigations.

This study has several limitations. The scRNA and snRNA datasets utilized in this research were produced by experts with proper, feasible controls. Nonetheless, the sample sizes were limited, which might be partly attributed to the technological limitations of single-cell analysis. Our analysis based on the limited sample sizes was likely associated with biases related to lifestyle, life stages, and environmental factors. These factors may impact MNTAg expression via multiple mechanisms, including protein stability. Fine particulate matter with particles with a diameter ≤ 2.5 µm (PM_2.5_) mean air pollution is a risk factor of MN [63]; in regions with PM_2.5_ > 70 µg/m^3^, each increase of 10 µg/m^3^ in PM_2.5_ concentration was accompanied with 14% higher odds for MN [63]. It thus remains a possibility that PM_2.5_ air pollution and other environmental factors may elevate MN risk via modulating MNTAg expression. The lack of analysis of MNTAg protein expression in this study is a limitation, which should be investigated in the future using comprehensive approaches, including proteomics, immunofluorescent/immunohistochemical staining, and Western Blotting. These approaches will further confirm MNTAg presence in podocytes, for instance, the co-staining of MNTAgs with established cell markers. The observations of this study should thus be interpreted with caution and should be properly validated in the future.

## 5. Conclusions

We provide the first analysis of multiple MNTAg expressions at the single-cell level in podocytes in the context of kidneys in adults, fetuses, and mice. (1) Among all these MNTAgs, PLA2R1 is expressed significantly more in adult human podocytes. (2) PLA2R1 displays a podocyte-specific expression within the kidney and can be regarded a podocyte marker. (3) PLA2R1 is a top-upregulated gene in the podocytes of CKD, AKI, and DN, indicating a general role of PLA2R1 in podocyte injury in kidney diseases associated with proteinuria. (4) PLA2R1 is not abundantly expressed in fetal podocytes, but SEMA3B does so, providing additional support for PLA2R1 and SEMA3B as adult and child MN target antigens, respectively. (5) Among the MNTAgs examined here, only Thsd7a displays podocyte-specific expression at a prominent level. This knowledge is useful when using mouse models for MN research.

## Figures and Tables

**Table 1:** PLA2R1 is a top-upregulated gene in the podocytes of CKD, AKI, and DN.

Disease	Gene	Log2fold	Effect Size	Adjusted *p*-Value
CKD	MAGI2	2.517	1.057	0
	PLA2R1	1.691	1.044	0
	PTPRQ	1.576	0.846	0
	NPAS3	1.445	0.913	0
	FTX	1.444	1.051	0
AKI	MAGI2	2.731	1.102	0
	PLA2R1	1.803	1.075	0
	PARD3B	1.603	1.022	0
	FTX	1.546	1.109	0
	DLG2	1.539	1.055	1.10 × 10^−272^
DN	MAGI2	3.713	1.447	0
	PTPRQ	2.885	1.493	7.53 × 10^−297^
	PLA2R1	2.586	1.498	0
	NPAS3	2.49	1.535	0
	PARD3B	2.417	1.515	0

Analyses were performed using the Differential Expression function within the CZ CELLxGENE Discover platform. The upregulated genes were produced via the comparisons of CKD (three datasets, 2946 podocytes) to normal (eleven datasets, 8257 podocytes), AKI (three datasets, 1471 podocytes) to normal, and DN (one dataset, 390 podocytes) to normal.

## Data Availability

All materials used in this study are either included in Supplementary Materials or available upon request.

## Supplementary Materials

The following supporting information can be downloaded at https://www.mdpi.com/article/10.3390/genes16030241/s1, Figure S1: Preference podocyte expression of PLA2R1 in healthy human kidney; Figure S2: Podocyte-specific expression of PLA2R1 in normal human kidney; Figure S3: Confirmation of the podocyte population using the podocyte marker NPHS1 in the indicated snRNAseq and scRNAseq datasets; Figure S4: Expression of PLA2R1 and the podocyte marker NPHS1 in podocytes; Figure S5: Expression of MN target antigens in normal human kidney; Figure S6: Expression of PLA2R1, NTNG1, HTRA1, and NDNF in situ within podocytes; Figure S7: Podocyte-specific expression of Thsd7a in mouse kidney; Figure S8: Expression of MN target antigens in fetal kidney; Figure S9: Expression of the indicated MN target antigens within fetal kidney; Table S1: Expression of MN target antigens in two human kidney scRNA datasets.

## Abbreviations

The following abbreviations are used in this manuscript:

AKI	Acute kidney injury
CKD	Chronic kidney disease
DN	Diabetic nephropathy
ESKD	End-stage kidney disease
GWAS	Genome-wide association studies
lncRNA	Long no-coding RNA
MN	Membranous nephropathy
MNTAgs	Membranous nephropathy target antigens
PMN	Primary MN
SMN	Secondary MN
scRNAseq	Single-cell RNA sequencing
snRNAseq	Single-nuclear RNA sequencing
SNPs	Single-nucleotide polymorphisms

## References

[1] Simon P., Ramee M.P., Autuly V., Laruelle E., Charasse C., Cam G., Ang K.S. (1994). Epidemiology of primary glomerular diseases in a french region. Variations according to period and age. Kidney Int..

[2] Maisonneuve P., Agodoa L., Gellert R., Stewart J.H., Buccianti G., Lowenfels A.B., Wolfe R.A., Jones E., Disney A.P., Briggs D. (2000). Distribution of primary renal diseases leading to end-stage renal failure in the united states, europe, and australia/new zealand: Results from an international comparative study. Am. J. Kidney Dis. Off. J. Natl. Kidney Found..

[3] Ronco P., Beck L., Debiec H., Fervenza F.C., Hou F.F., Jha V., Sethi S., Tong A., Vivarelli M., Wetzels J. (2021). Membranous nephropathy. Nat. Rev. Dis. Primers.

[4] Jha V., Ganguli A., Saha T.K., Kohli H.S., Sud K., Gupta K.L., Joshi K., Sakhuja V. (2007). A randomized, controlled trial of steroids and cyclophosphamide in adults with nephrotic syndrome caused by idiopathic membranous nephropathy. J. Am. Soc. Nephrol. JASN.

[5] Ponticelli C., Zucchelli P., Passerini P., Cesana B., Locatelli F., Pasquali S., Sasdelli M., Redaelli B., Grassi C., Pozzi C. (1995). A 10-year follow-up of a randomized study with methylprednisolone and chlorambucil in membranous nephropathy. Kidney Int..

[6] Heymann W., Hackel D.B., Harwood S., Wilson S.G., Hunter J.L. (1959). Production of nephrotic syndrome in rats by freund’s adjuvants and rat kidney suspensions. Proc. Soc. Exp. Biol. Med..

[7] Kerjaschki D., Farquhar M.G. (1982). The pathogenic antigen of heymann nephritis is a membrane glycoprotein of the renal proximal tubule brush border. Proc. Natl. Acad. Sci. USA.

[8] Beck L.H., Bonegio R.G., Lambeau G., Beck D.M., Powell D.W., Cummins T.D., Klein J.B., Salant D.J. (2009). M-type phospholipase a2 receptor as target antigen in idiopathic membranous nephropathy. N. Engl. J. Med..

[9] Kerjaschki D., Farquhar M.G. (1983). Immunocytochemical localization of the heymann nephritis antigen (gp330) in glomerular epithelial cells of normal lewis rats. J. Exp. Med..

[10] Tomas N.M., Beck L.H., Meyer-Schwesinger C., Seitz-Polski B., Ma H., Zahner G., Dolla G., Hoxha E., Helmchen U., Dabert-Gay A.S. (2014). Thrombospondin type-1 domain-containing 7a in idiopathic membranous nephropathy. N. Engl. J. Med..

[11] Reinhard L., Machalitza M., Wiech T., Grone H.J., Lasse M., Rinschen M.M., Ferru N., Brasen J.H., Dromann F., Rob P.M. (2022). Netrin g1 is a novel target antigen in primary membranous nephropathy. J. Am. Soc. Nephrol. JASN.

[12] Caza T.N., Hassen S.I., Kenan D.J., Storey A., Arthur J.M., Herzog C., Edmondson R.D., Bourne T.D., Beck L.H., Larsen C.P. (2021). Transforming growth factor beta receptor 3 (tgfbr3)-associated membranous nephropathy. Kidney360.

[13] Laith Al-Rabadi T.C., Avillach C., Aylin R., Rodan B., Williams J., Abraham M., Revelo Penafie P., Nicole K., Andeen I., Kawalit F.C. High Temperature Recombinant Protein a1 (htra1): A Novel Antigen in Membranous Nephropathy. Proceedings of the 2020 ASN Kidney Week.

[14] Sethi S., Madden B., Casal Moura M., Singh R.D., Nasr S.H., Hou J., Sharma A., Nath K.A., Specks U., Fervenza F.C. (2023). Membranous nephropathy in syphilis is associated with neuron-derived neurotrophic factor. J. Am. Soc. Nephrol. JASN.

[15] Sethi S., Debiec H., Madden B., Vivarelli M., Charlesworth M.C., Ravindran A., Gross L., Ulinski T., Buob D., Tran C.L. (2020). Semaphorin 3b-associated membranous nephropathy is a distinct type of disease predominantly present in pediatric patients. Kidney Int..

[16] Sethi S., Madden B., Casal Moura M., Nasr S.H., Klomjit N., Gross L., Negron V., Charlesworth M.C., Alexander M.P., Leung N. (2022). Hematopoietic stem cell transplant-membranous nephropathy is associated with protocadherin fat1. J. Am. Soc. Nephrol. JASN.

[17] Sethi S., Madden B.J., Debiec H., Charlesworth M.C., Gross L., Ravindran A., Hummel A.M., Specks U., Fervenza F.C., Ronco P. (2019). Exostosin 1/exostosin 2-associated membranous nephropathy. J. Am. Soc. Nephrol. JASN.

[18] Le Quintrec M., Teisseyre M., Bec N., Delmont E., Szwarc I., Perrochia H., Machet M.C., Chauvin A., Mavroudakis N., Taieb G. (2021). Contactin-1 is a novel target antigen in membranous nephropathy associated with chronic inflammatory demyelinating polyneuropathy. Kidney Int..

[19] Sethi S., Debiec H., Madden B., Charlesworth M.C., Morelle J., Gross L., Ravindran A., Buob D., Jadoul M., Fervenza F.C. (2020). Neural epidermal growth factor-like 1 protein (nell-1) associated membranous nephropathy. Kidney Int..

[20] Sanjeev Sethi B.J.M., Gross L., Negron V.C., Charlesworth C., Debiec H., Ronco P.M., Fervenza F.C. Protocadherin 7-associated membranous nephropathy. Proceedings of the 2020 ASN Kidney Week.

[21] Sethi S., Casal Moura M., Madden B., Debiec H., Nasr S.H., Larsen C.P., Gross L., Negron V., Singh R.D., Nath K.A. (2023). Proprotein convertase subtilisin/kexin type 6 (pcsk6) is a likely antigenic target in membranous nephropathy and nonsteroidal anti-inflammatory drug use. Kidney Int..

[22] Caza T.N., Hassen S.I., Kuperman M., Sharma S.G., Dvanajscak Z., Arthur J., Edmondson R., Storey A., Herzog C., Kenan D.J. (2020). Neural cell adhesion molecule 1 is a novel autoantigen in membranous lupus nephritis. Kidney Int..

[23] Gu Y., Xu H., Tang D. (2021). Mechanisms of primary membranous nephropathy. Biomolecules.

[24] Avasare R., Andeen N., Beck L. (2024). Novel antigens and clinical updates in membranous nephropathy. Ann. Rev. Med..

[25] Fehmi J., Davies A.J., Antonelou M., Keddie S., Pikkupeura S., Querol L., Delmont E., Cortese A., Franciotta D., Persson S. (2023). Contactin-1 links autoimmune neuropathy and membranous glomerulonephritis. PLoS ONE.

[26] Van de Logt A.E., Fresquet M., Wetzels J.F., Brenchley P. (2019). The anti-pla2r antibody in membranous nephropathy: What we know and what remains a decade after its discovery. Kidney Int..

[27] Radhakrishnan Y., Zand L., Sethi S., Fervenza F.C. (2024). Membranous nephropathy treatment standard. Nephrol. Dial. Transplant. Off. Publ. Eur. Dial. Transpl. Assoc. Eur. Ren. Assoc..

[28] Program C.Z.I.C.S., Abdulla S., Aevermann B., Assis P., Badajoz S., Bell S.M., Bezzi E., Cakir B., Chaffer J., Chambers S. (2025). Cz cellxgene discover: A single-cell data platform for scalable exploration, analysis and modeling of aggregated data. Nucleic Acids Res..

[29] Lake B.B., Menon R., Winfree S., Hu Q., Melo Ferreira R., Kalhor K., Barwinska D., Otto E.A., Ferkowicz M., Diep D. (2023). An atlas of healthy and injured cell states and niches in the human kidney. Nature.

[30] Xu C., Prete M., Webb S., Jardine L., Stewart B.J., Hoo R., He P., Meyer K.B., Teichmann S.A. (2023). Automatic cell-type harmonization and integration across human cell atlas datasets. Cell.

[31] McEvoy C.M., Murphy J.M., Zhang L., Clotet-Freixas S., Mathews J.A., An J., Karimzadeh M., Pouyabahar D., Su S., Zaslaver O. (2022). Single-cell profiling of healthy human kidney reveals features of sex-based transcriptional programs and tissue-specific immunity. Nat. Commun..

[32] Wilson P.C., Muto Y., Wu H., Karihaloo A., Waikar S.S., Humphreys B.D. (2022). Multimodal single cell sequencing implicates chromatin accessibility and genetic background in diabetic kidney disease progression. Nat. Commun..

[33] Marshall J.L., Noel T., Wang Q.S., Chen H., Murray E., Subramanian A., Vernon K.A., Bazua-Valenti S., Liguori K., Keller K. (2022). High-resolution slide-seqv2 spatial transcriptomics enables discovery of disease-specific cell neighborhoods and pathways. iScience.

[34] Muto Y., Wilson P.C., Ledru N., Wu H., Dimke H., Waikar S.S., Humphreys B.D. (2021). Single cell transcriptional and chromatin accessibility profiling redefine cellular heterogeneity in the adult human kidney. Nat. Commun..

[35] Stewart B.J., Ferdinand J.R., Young M.D., Mitchell T.J., Loudon K.W., Riding A.M., Richoz N., Frazer G.L., Staniforth J.U.L., Vieira Braga F.A. (2019). Spatiotemporal immune zonation of the human kidney. Science.

[36] Young M.D., Mitchell T.J., Vieira Braga F.A., Tran M.G.B., Stewart B.J., Ferdinand J.R., Collord G., Botting R.A., Popescu D.M., Loudon K.W. (2018). Single-cell transcriptomes from human kidneys reveal the cellular identity of renal tumors. Science.

[37] Novella-Rausell C., Grudniewska M., Peters D.J.M., Mahfouz A. (2023). A comprehensive mouse kidney atlas enables rare cell population characterization and robust marker discovery. iScience.

[38] Ancian P., Lambeau G., Mattei M.G., Lazdunski M. (1995). The human 180-kda receptor for secretory phospholipases a2. Molecular cloning, identification of a secreted soluble form, expression, and chromosomal localization. J. Biol. Chem..

[39] Boute N., Gribouval O., Roselli S., Benessy F., Lee H., Fuchshuber A., Dahan K., Gubler M.C., Niaudet P., Antignac C. (2000). Nphs2, encoding the glomerular protein podocin, is mutated in autosomal recessive steroid-resistant nephrotic syndrome. Nat. Genet..

[40] Kestila M., Lenkkeri U., Mannikko M., Lamerdin J., McCready P., Putaala H., Ruotsalainen V., Morita T., Nissinen M., Herva R. (1998). Positionally cloned gene for a novel glomerular protein--nephrin--is mutated in congenital nephrotic syndrome. Mol. Cell.

[41] Kann M., Ettou S., Jung Y.L., Lenz M.O., Taglienti M.E., Park P.J., Schermer B., Benzing T., Kreidberg J.A. (2015). Genome-wide analysis of wilms’ tumor 1-controlled gene expression in podocytes reveals key regulatory mechanisms. J. Am. Soc. Nephrol. JASN.

[42] Uchida T., Oda T. (2024). The prevalence, characteristics, and putative mechanisms of dual antigen-positive membranous nephropathy: The underestimated condition. Int. J. Mol. Sci..

[43] Bharati J., Waguespack D.R., Beck L.H. (2024). Membranous nephropathy: Updates on management. Adv. Kidney Dis. Health.

[44] Tomas N.M., Hoxha E., Reinicke A.T., Fester L., Helmchen U., Gerth J., Bachmann F., Budde K., Koch-Nolte F., Zahner G. (2016). Autoantibodies against thrombospondin type 1 domain-containing 7a induce membranous nephropathy. J. Clin. Investig..

[45] Georgas K., Rumballe B., Valerius M.T., Chiu H.S., Thiagarajan R.D., Lesieur E., Aronow B.J., Brunskill E.W., Combes A.N., Tang D. (2009). Analysis of early nephron patterning reveals a role for distal rv proliferation in fusion to the ureteric tip via a cap mesenchyme-derived connecting segment. Dev. Biol..

[46] Schnabel E., Dekan G., Miettinen A., Farquhar M.G. (1989). Biogenesis of podocalyxin—The major glomerular sialoglycoprotein—In the newborn rat kidney. Eur. J. Cell Biol..

[47] Schnabel E., Anderson J.M., Farquhar M.G. (1990). The tight junction protein zo-1 is concentrated along slit diaphragms of the glomerular epithelium. J. Cell Biol..

[48] Mundlos S., Pelletier J., Darveau A., Bachmann M., Winterpacht A., Zabel B. (1993). Nuclear localization of the protein encoded by the wilms’ tumor gene wt1 in embryonic and adult tissues. Development.

[49] Greka A., Mundel P. (2012). Cell biology and pathology of podocytes. Ann. Rev. Physiol..

[50] Kreidberg J.A., Sariola H., Loring J.M., Maeda M., Pelletier J., Housman D., Jaenisch R. (1993). Wt-1 is required for early kidney development. Cell.

[51] Somlo S., Mundel P. (2000). Getting a foothold in nephrotic syndrome. Nat. Genet..

[52] Kerjaschki D. (2001). Caught flat-footed: Podocyte damage and the molecular bases of focal glomerulosclerosis. J. Clin. Investig..

[53] Jiang H., Shen Z., Zhuang J., Lu C., Qu Y., Xu C., Yang S., Tian X. (2023). Understanding the podocyte immune responses in proteinuric kidney diseases: From pathogenesis to therapy. Front. Immunol..

[54] Kriz W., Gretz N., Lemley K.V. (1998). Progression of glomerular diseases: Is the podocyte the culprit?. Kidney Int..

[55] Basile D.P., Donohoe D., Roethe K., Osborn J.L. (2001). Renal ischemic injury results in permanent damage to peritubular capillaries and influences long-term function. Am. J. Physiol. Ren. Physiol..

[56] Parr S.K., Matheny M.E., Abdel-Kader K., Greevy R.A., Bian A., Fly J., Chen G., Speroff T., Hung A.M., Ikizler T.A. (2018). Acute kidney injury is a risk factor for subsequent proteinuria. Kidney Int..

[57] Ashraf S., Kudo H., Rao J., Kikuchi A., Widmeier E., Lawson J.A., Tan W., Hermle T., Warejko J.K., Shril S. (2018). Mutations in six nephrosis genes delineate a pathogenic pathway amenable to treatment. Nat. Commun..

[58] Chen Z., Zhang M., Lu Y., Ding T., Liu Z., Liu Y., Zhou Z., Wang L. (2022). Overexpressed lncrna ftx promotes the cell viability, proliferation, migration and invasion of renal cell carcinoma via ftx/mir-4429/ube2c axis. Oncol. Rep..

[59] Koehler S., Tellkamp F., Niessen C.M., Bloch W., Kerjaschki D., Schermer B., Benzing T., Brinkkoetter P.T. (2016). Par3a is dispensable for the function of the glomerular filtration barrier of the kidney. Am. J. Physiol. Ren. Physiol..

[60] Sethi S., Beck L.H., Glassock R.J., Haas M., De Vriese A.S., Caza T.N., Hoxha E., Lambeau G., Tomas N.M., Madden B. (2023). Mayo clinic consensus report on membranous nephropathy: Proposal for a novel classification. Kidney Int..

[61] Stanescu H.C., Arcos-Burgos M., Medlar A., Bockenhauer D., Kottgen A., Dragomirescu L., Voinescu C., Patel N., Pearce K., Hubank M. (2011). Risk hla-dqa1 and pla(2)r1 alleles in idiopathic membranous nephropathy. N. Engl. J. Med..

[62] Hanasaki K., Yokota Y., Ishizaki J., Itoh T., Arita H. (1997). Resistance to endotoxic shock in phospholipase a2 receptor-deficient mice. J. Biol. Chem..

[63] Xu X., Wang G., Chen N., Lu T., Nie S., Xu G., Zhang P., Luo Y., Wang Y., Wang X. (2016). Long-term exposure to air pollution and increased risk of membranous nephropathy in China. J. Am. Soc. Nephrol. JASN.

