# Supplementary material for: Membranous Nephropathy Target Antigens Display Podocyte-Specific and Non-Specific Expression in Healthy Kidneys
Source: Genes (Basel). 2025 Feb 20;16(3):241. doi: 10.3390/genes16030241 (PMC11942440; doi:10.3390/genes16030241)
Supplement: Supplementary file 1 [file genes-16-00241-s001.zip › Sup Figs S1-S9.pdf]

Supplementary Figure S1

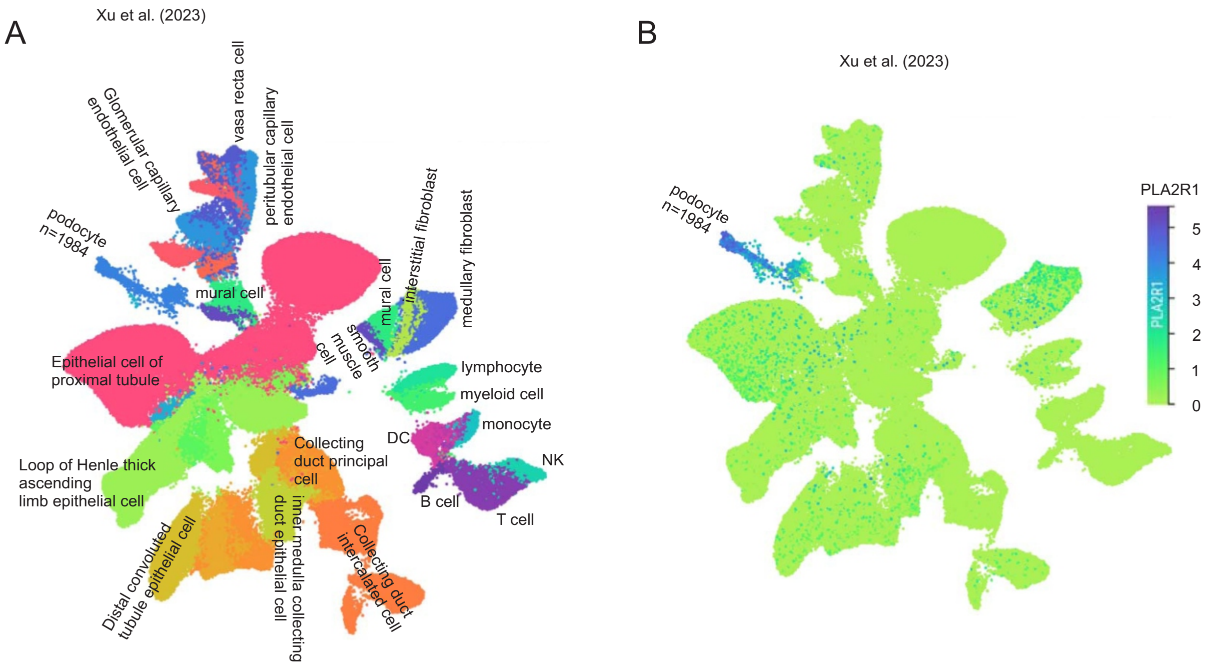

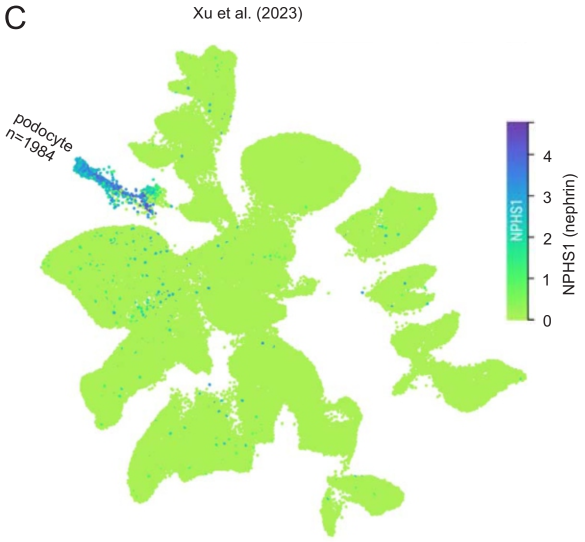

Supplementary Figure S1. Preference podocyte expression of PLA2R1 in healthy human kidney. The Xu et al (2023) (reference 30) single-cell dataset derived from n=5 human kidneys within the CZ CELLxGENE Discover platform (https://cellxgene.cziscience.com/) (reference 28) was used in the analysis. (**A**) Map of kidney cells. (**B, C**) Expression of PLA2R1 (B) and the podocyte marker nephrin encoded by the *NPHS1* gene (C) in individual kidney cell populations. The podocyte cluster and the number of podocytes within the cluster are indicated.

Supplementary Figure S2

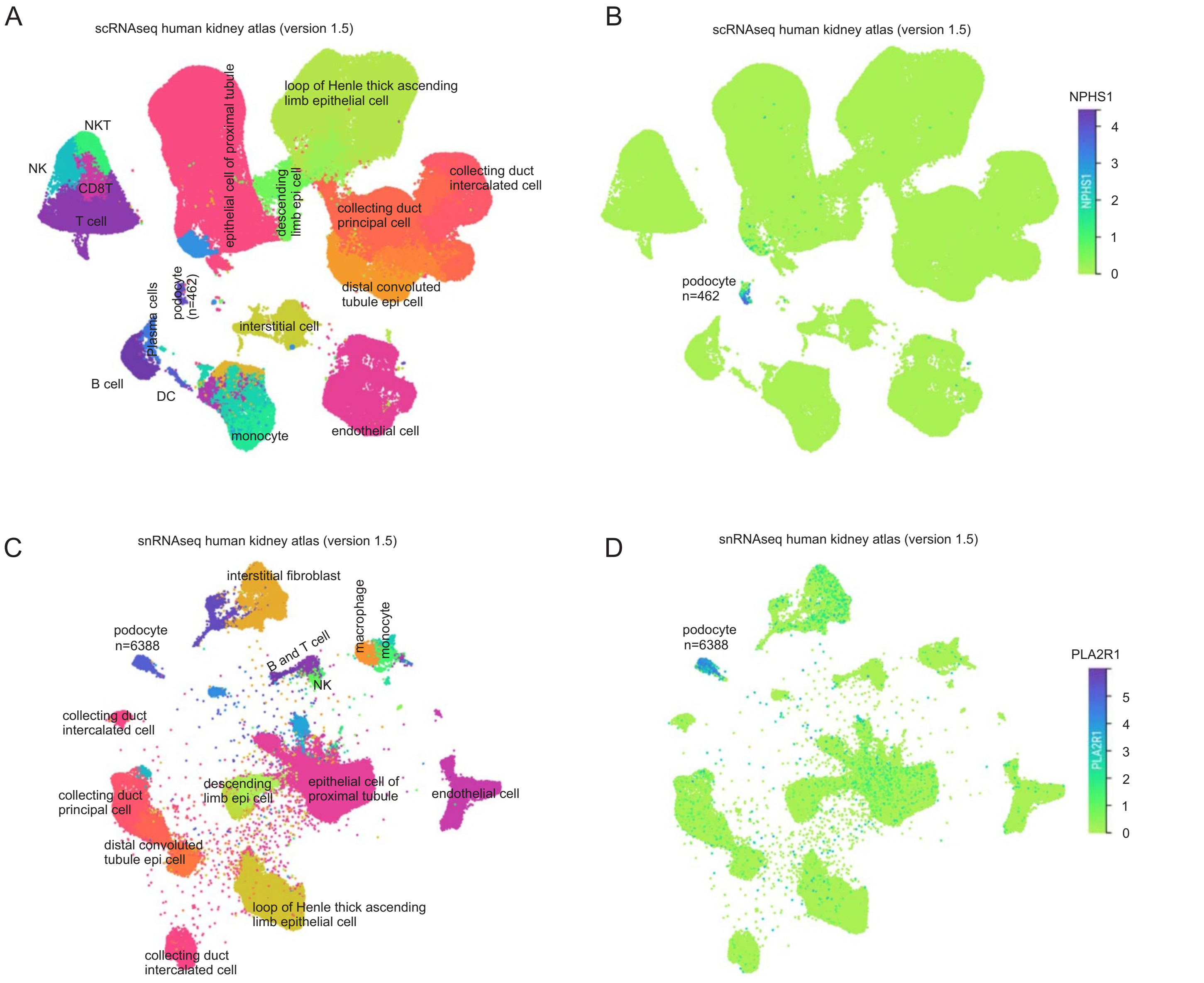

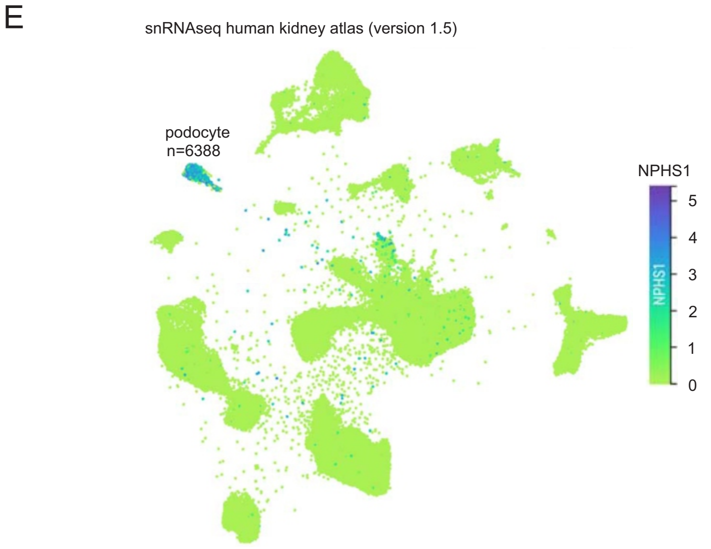

Supplementary Figure S2. Podocyte-specific expression of PLA2R1 in normal human kidney. The single-cell RNAseq (scRNAseq) and single-nuclear RNAseq (snRNAseq) datasets of human kidney atlas version 1.5 within the CZ CELLxGENE Discover were utilized in the analyses. Both datasets were derived from the study by Laket et al. (2023) (reference 29) and contained 4 tissues. (**A, C**) Maps of cell populations in the indicated datasets. (**B, D, E**) The presence of established podocyte marker NPHS1 and PLA2R1 in the indicated datasets. The podocyte cluster and the number of podocytes within the cluster are marked.

Supplementary Figure S3

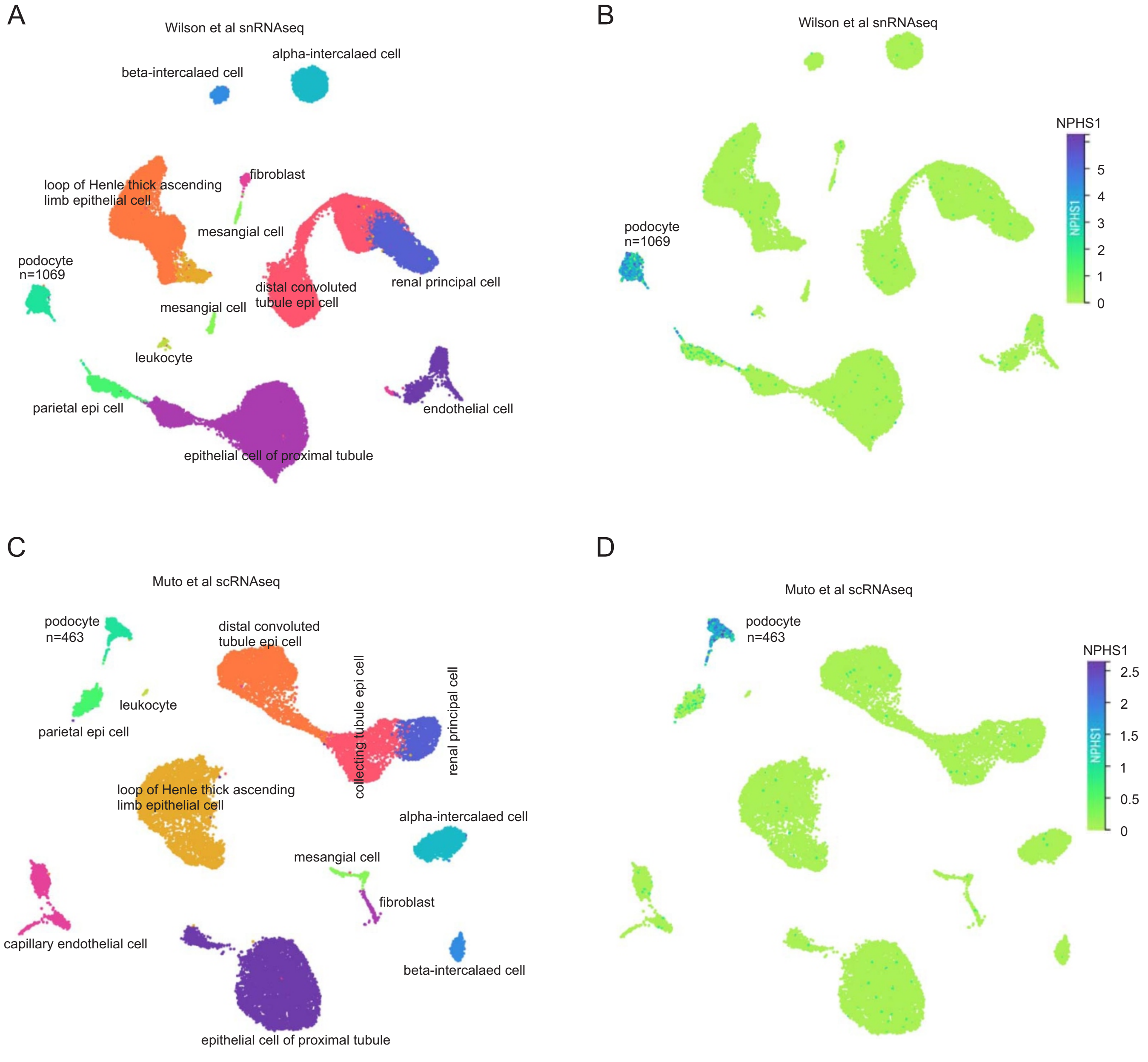

Supplementary Figure S3. Confirmation of the podocyte population using the podocyte marker NPHS1 in the indicated snRNAseq and scRNAseq datasets. Both datasets were organized by CZ CELLxGENE Discover.

Supplementary Figure S4

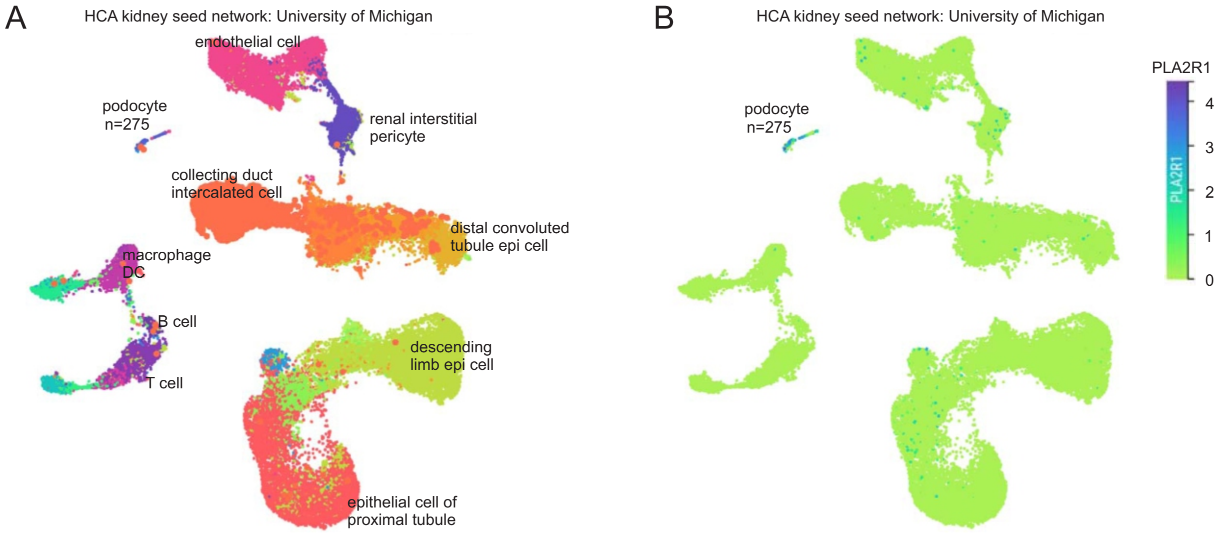

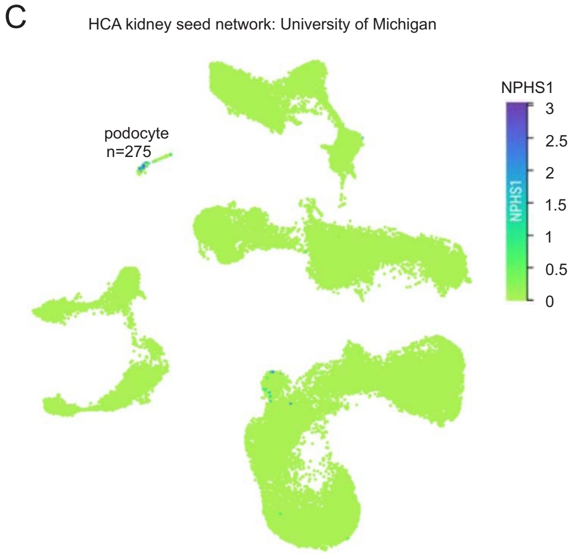

Supplementary Figure S4. Expression of PLA2R1 and the podocyte marker NPHS1 in podocytes. The scRNAseq dataset of HCK kidney seed network: University of Michigan within CZ CELLxGENE Discover was used for the analysis. **A**) Map of kidney cells. (**B, C**) Expression of PLA2R1 (B) and NPHS1 (C) in individual kidney cell populations with the podocyte cluster indicated.

Supplementary Figure S5

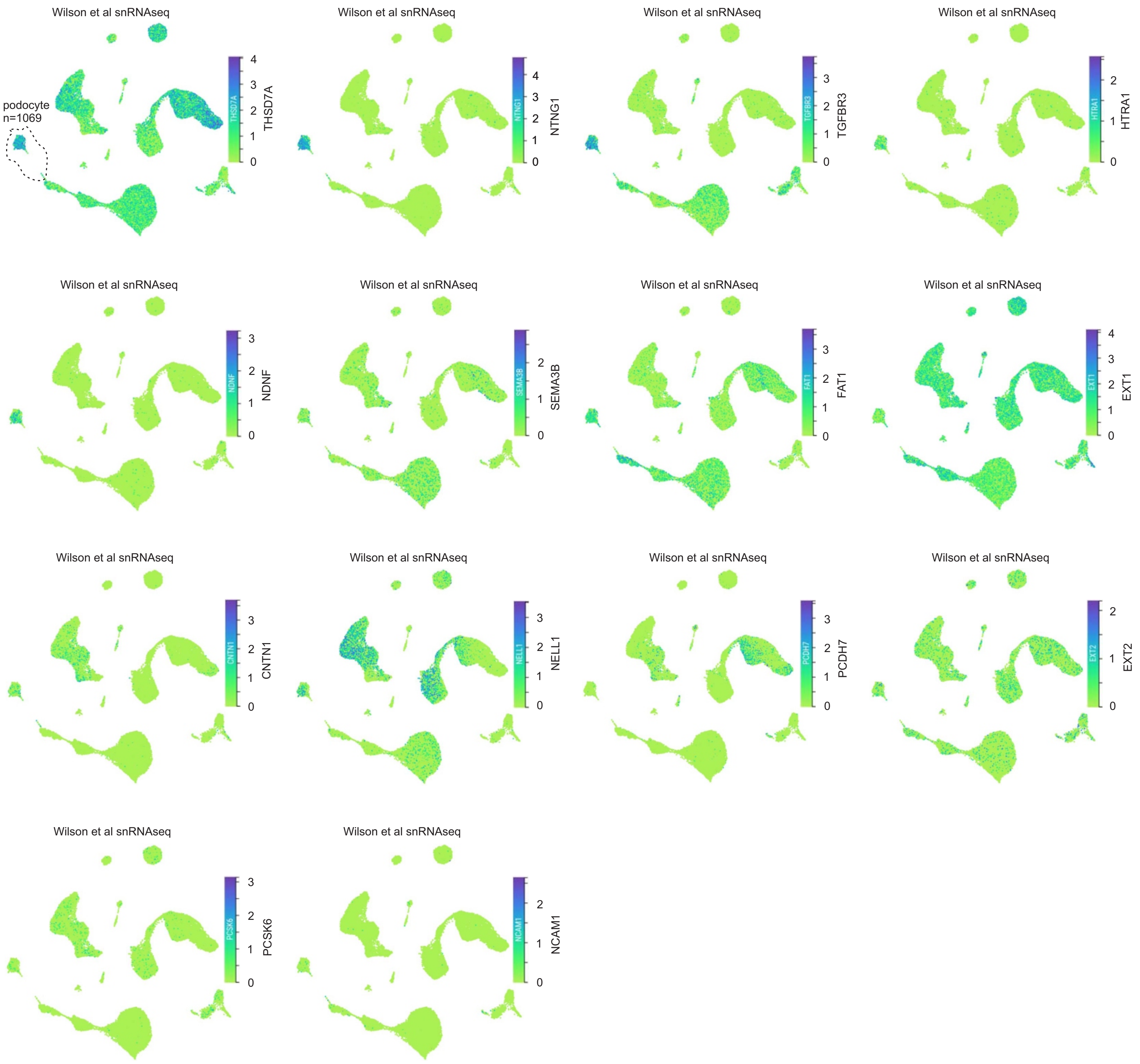

Supplementary Figure S5. Expression of MN target antigens in normal human kidney. The Wilson snRNAseq dataset (reference 32) within CZ CELLxGENE Discover was used to analyze the expression of THSD7A, NTNG1, TGFBR3, HTRA1, NDNF, SEMA3B, FAT1, EXT1, CNTN1, NELL1, PCDH7, EXT2, PCSK6, and NCAM1. The podocyte cluster and the number of cells in the cluster are indicated. Please see Supplementary Figure S3A for the identify of individual cell clusters and Supplementary Figure S3B for the confirmation of the podocyte cluster using NPHS1.

Supplementary Figure S6

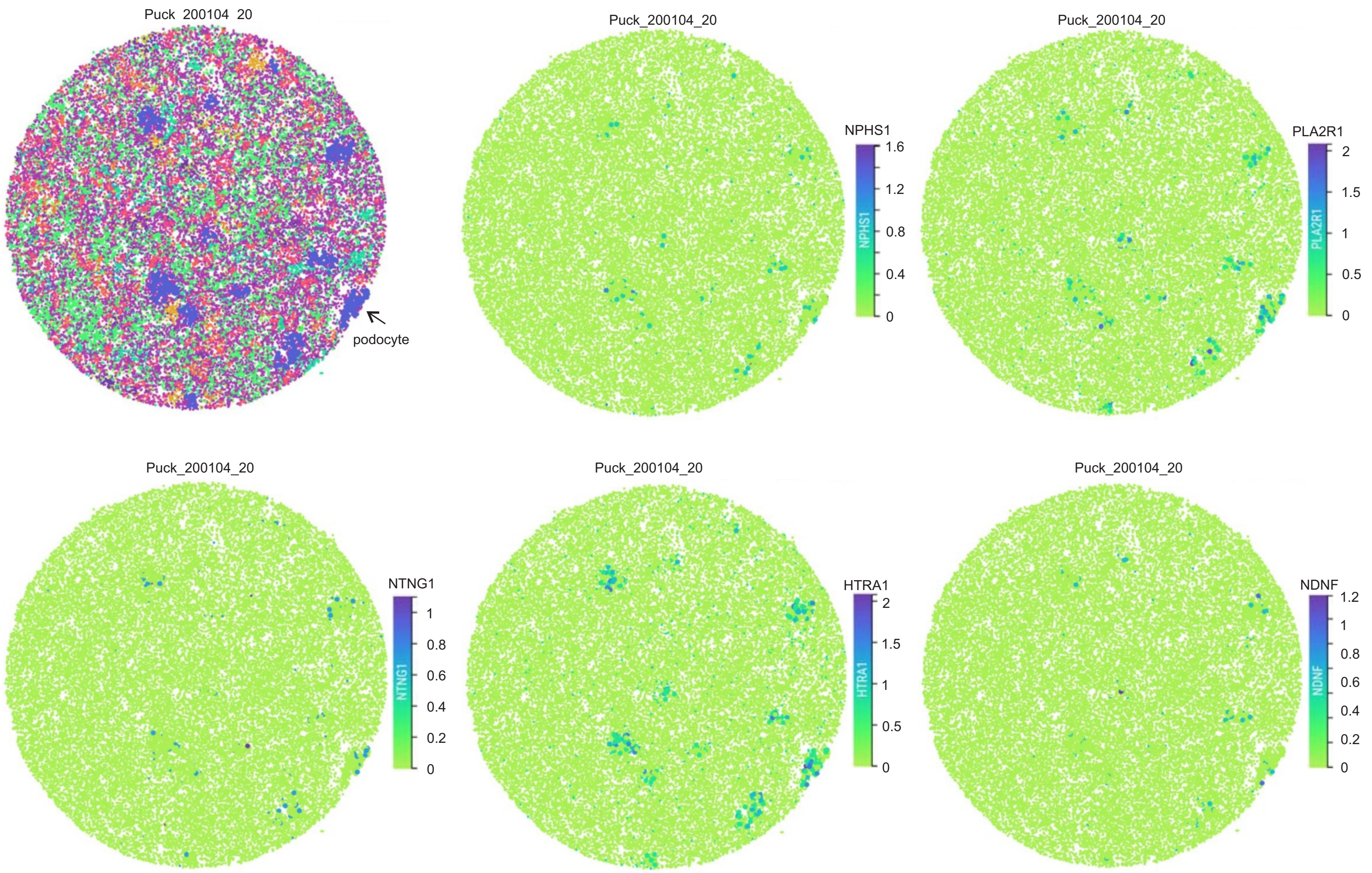

Supplementary Figure S6. Expression of PLA2R1, NTNG1, HTRA1, and NDNF in situ within podocytes. The Marshall et al. (2022) dataset (reference 33) was used in the analysis. Top left image: cell map of human kidney tissue in the slide Puck_200204_20. The expression of the podocyte marker NHPS1, PLA2R1, NTNG1, HTRA1, and NDDF is shown.

Supplementary Figure S7

A

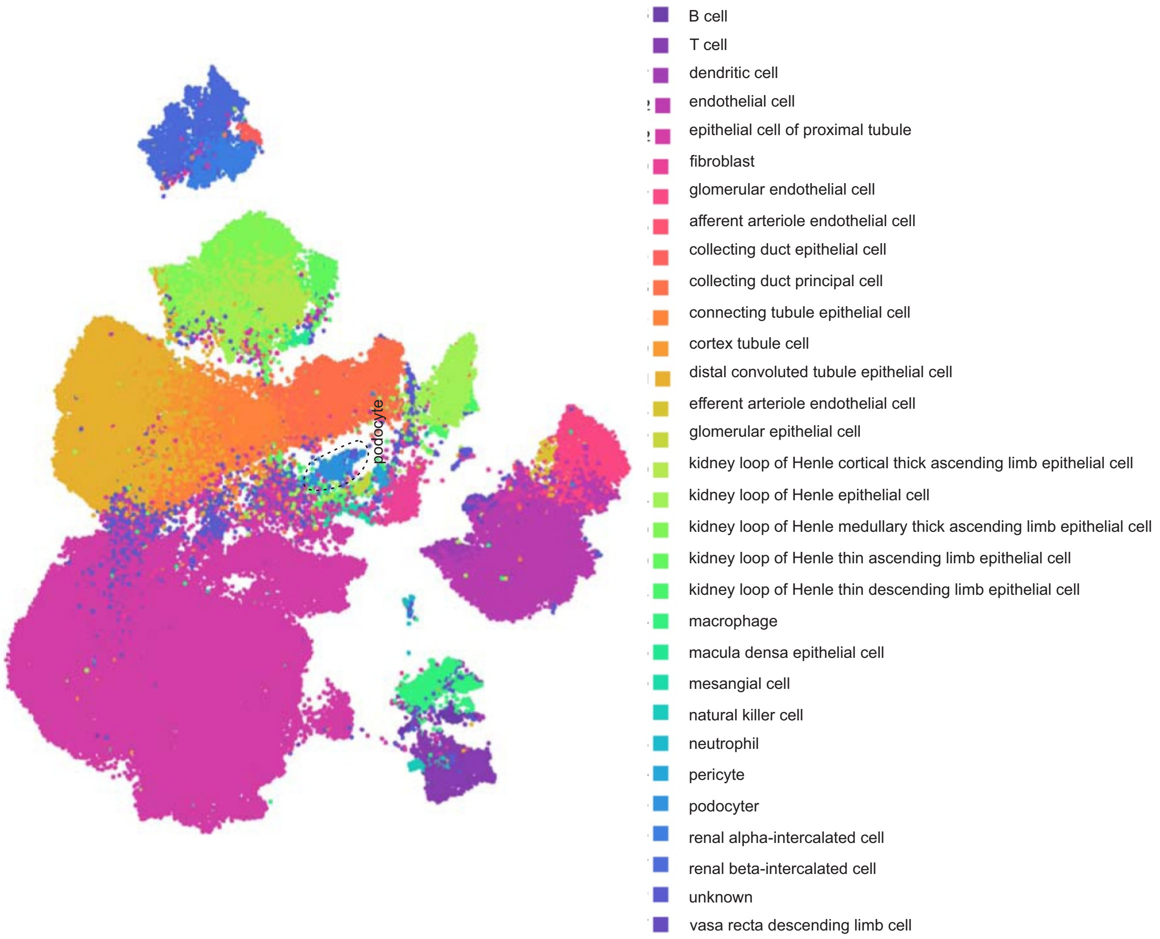

B

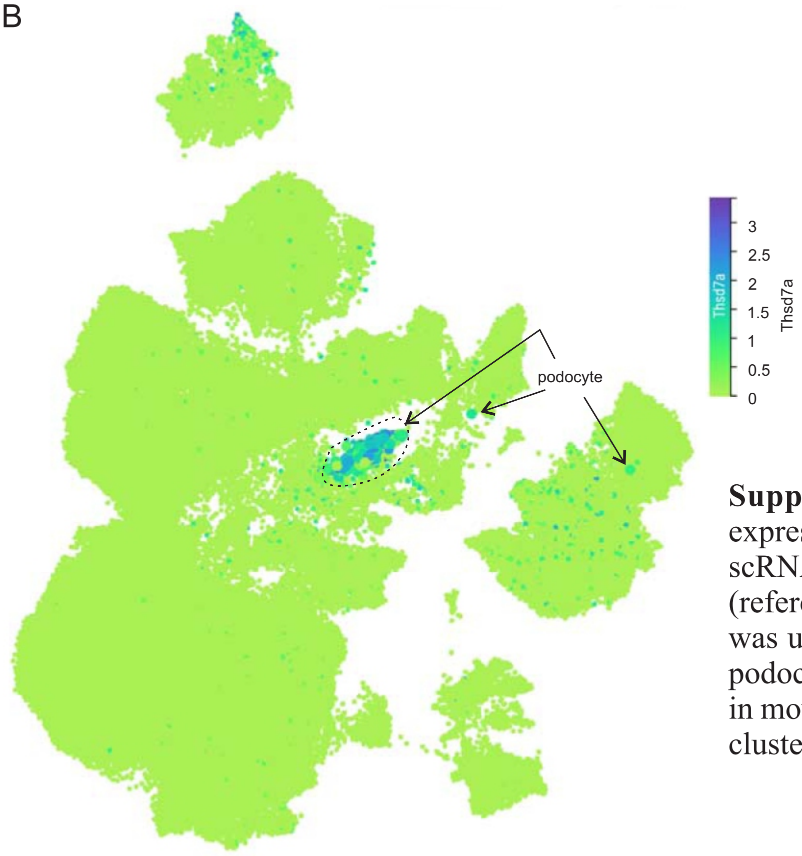

Supplementary Figure S7. Podocyte-specific expression of Thsd7a in mouse kidney. The mouse scRNAseq dataset by Novella-Rausell et al. (2023) (reference 37) within CZ CELLxGENE Discover was used for the analysis. (**A**) Single cell map; the podocyte cluster is marked. (**B**) Thsd7a expression in mouse kidney cell populations with the podocyte cluster and individual podocytes indicated.

Supplementary Figure S8

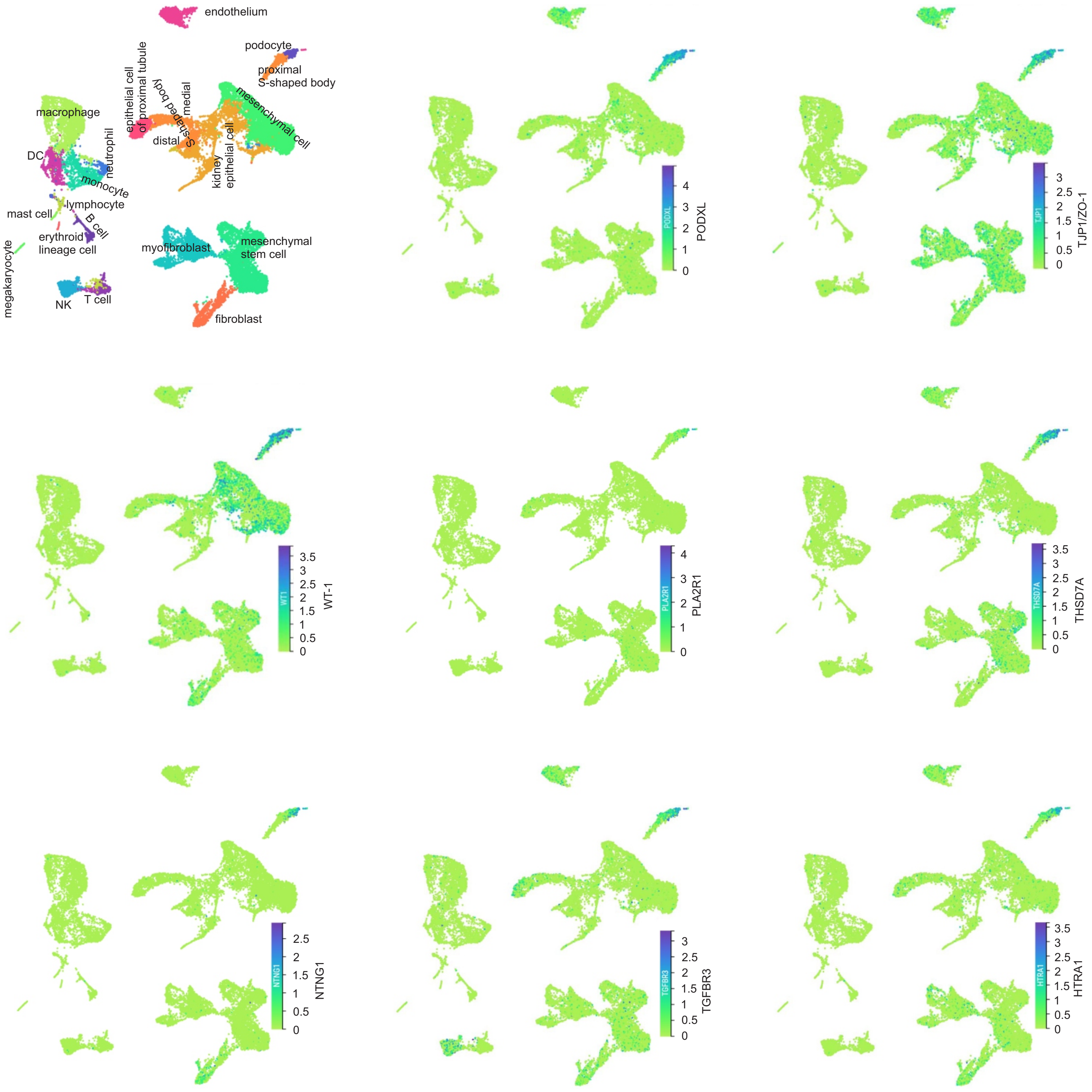

Supplementary Figure S8. Expression of MN target antigens in fetal kidney. Fetal kidney scRNAseq dataset by Stewart et al (2019) (reference 35) within CZ CELLxGENE Discover was used for the analysis. Top left panel: the standard single-cell map was used; the identities of proximal, medial, and distal S-shaped body were by the authors. PODXL, TJP1, and WT-1 were used as positive controls for immature podocytes within the proximal S-shaped body and mature podocytes. The expression of the indicated MN target antigens within fetal kidney is shown.

Supplementary Figure S9

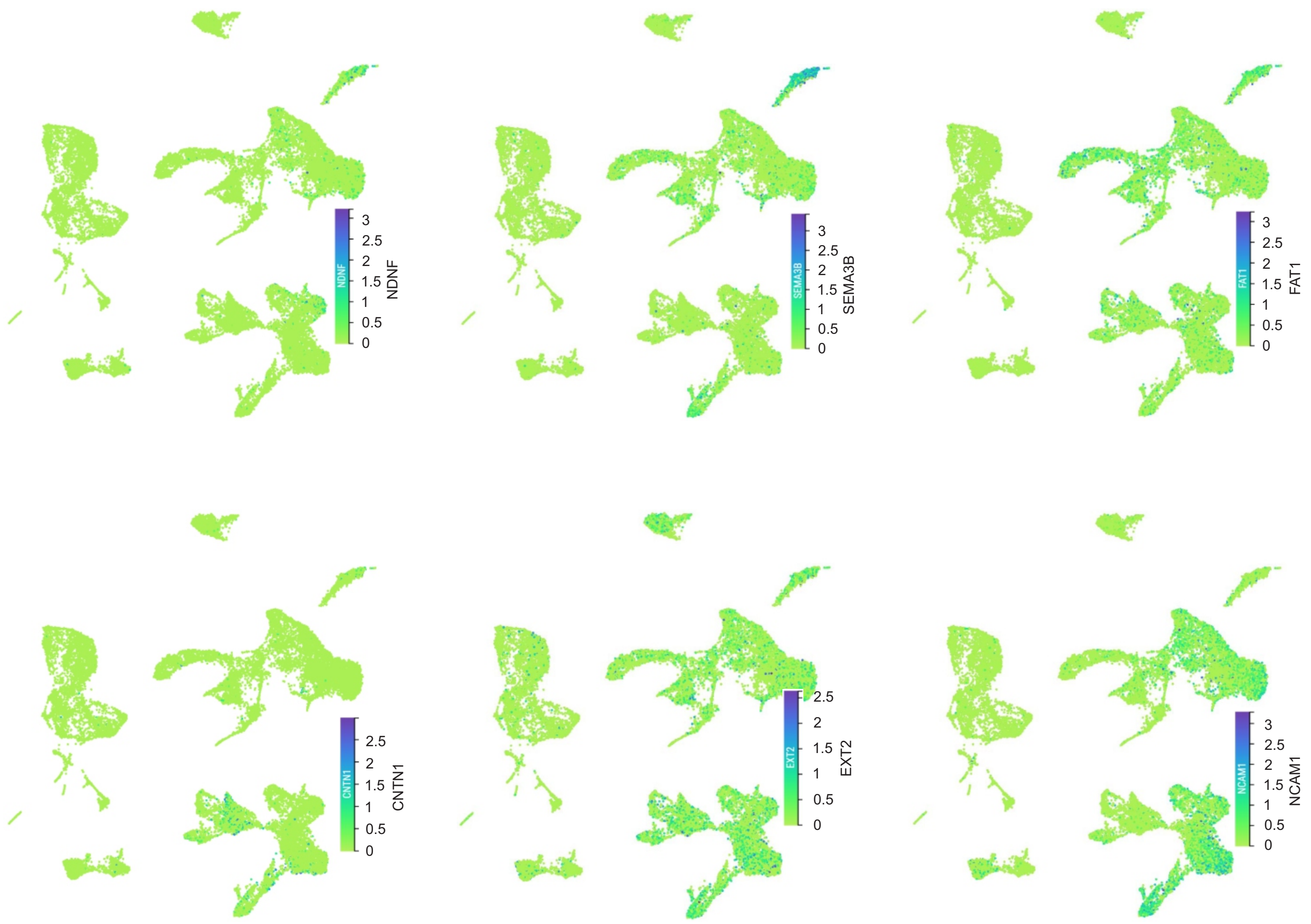

Supplementary Figure S9. Expression of the indicated MN target antigens within fetal kidney. This figure is the continuation of Supplementary Figure 8.
